# Mapping Arbovirus-Vector Interactions Using Systems Biology Techniques

**DOI:** 10.3389/fcimb.2018.00440

**Published:** 2019-01-07

**Authors:** Marine J. Petit, Priya S. Shah

**Affiliations:** ^1^Department of Microbiology and Molecular Genetics, University of California, Davis, Davis, CA, United States; ^2^Department of Chemical Engineering, University of California, Davis, Davis, CA, United States

**Keywords:** arbovirus, virus, arthropod, vector, interactions, systems biology

## Abstract

Studying how arthropod-borne viruses interact with their arthropod vectors is critical to understanding how these viruses replicate and are transmitted. Until recently, these types of studies were limited in scale because of the lack of classical tools available to study virus-host interaction for non-model viruses and non-model organisms. Advances in systems biology “-omics”-based techniques such as next-generation sequencing (NGS) and mass spectrometry can rapidly provide an unbiased view of arbovirus-vector interaction landscapes. In this mini-review, we discuss how arbovirus-vector interaction studies have been advanced by systems biology. We review studies of arbovirus-vector interactions that occur at multiple time and length scales, including intracellular interactions, interactions at the level of the organism, viral and vector populations, and how new techniques can integrate systems-level data across these different scales.

## Introduction

Arthropod-borne viruses (arboviruses), which are transmitted by arthropod vectors like mosquitoes, flies, and ticks, are a source of endemic, emerging, and re-emerging infectious diseases. Chikungunya virus (CHIKV), a mosquito-borne virus, causes severe arthritic disease with over one million estimated infections in a single epidemic (Powers and Logue, 2007). Mosquito-borne dengue virus (DENV) infects nearly 400 million people annually (Bhatt et al., 2013) and can cause severe illness such as dengue hemorrhagic fever and dengue shock syndrome (Gubler, 2002). Zika virus (ZIKV) is a recently emerged mosquito-borne virus that causes major developmental defects when fetuses are infected *in utero* (Mlakar et al., 2016; Delaney et al., 2018). Tick-borne viruses, including Powassan virus, can also be neuropathogenic and are increasing in prevalence. As habitats for arthropod vectors expand with global climate change, residents of densely-populated regions will be at risk of arbovirus infections.

**Figure 1:**
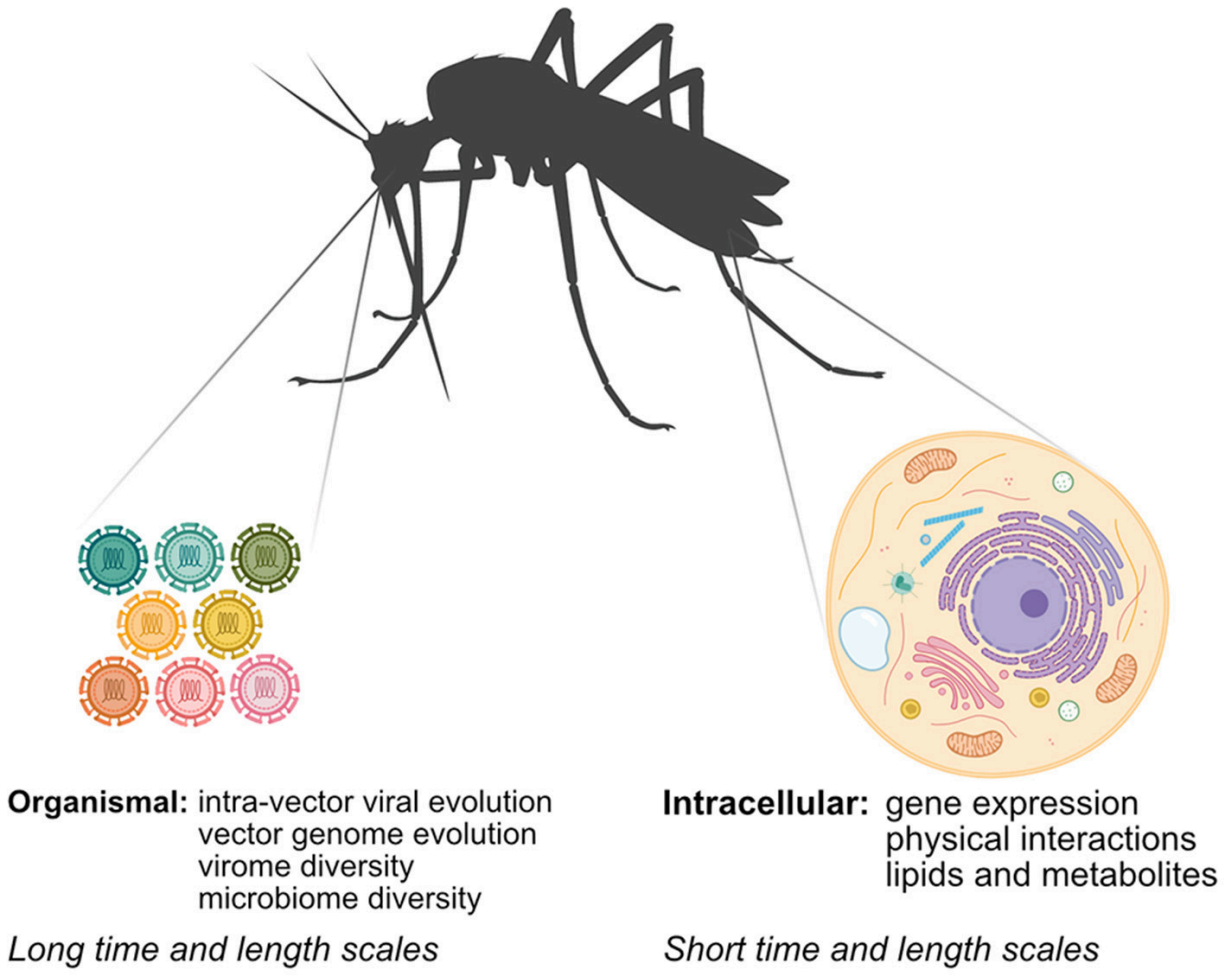
Mapping arbovirus-vector interactions at different scales using systems biology. Arboviruses interact with their vectors at the intracellular and organismal scale. Multiple–omics techniques can be used at the intracellular scale, while interactions at the organismal scale rely primarily on NGS techniques.

Unraveling how arboviruses interact with their vectors is critical to understanding arbovirus replication and transmission and informing arbovirus mitigation strategies. While classical methods have provided significant insight into arbovirus-vector interactions, systems biology approaches, which serve to generate and integrate large unbiased datasets using “-omics”-based approaches, have several advantages. First, systems biology approaches offer an unbiased view of arbovirus-vector interactions, leading to discoveries that may not have been possible using hypothesis-driven approaches. Second, researchers can use technologies like next-generation sequencing (NGS) and mass spectrometry (MS) to directly sample vector nucleic acids and proteins to answer scientific questions that were previously intractable in non-model systems that lack many classical genetic or biochemical tools. Finally, system approaches enable researchers to rapidly map arbovirus-vector interactions of newly emerging arboviruses. In this mini-review, we explore how systems biology approaches have been applied to study aspects of arbovirus-vector interactions at different scales (Figure 1). We review advances in identifying intracellular arbovirus-vector interactions that occur in response to infection. We also discuss how advances in NGS methods can provide insight into interactions occurring over larger time and length scales, and can be used to bridge arbovirus-vector interactions occurring over multiple scales. Finally, we consider how specific challenges in studying arbovirus-vector interactions may be addressed in the future.

## Identifying Intracellular Arbovirus-vector Interactions

All viruses must hijack host machinery and resources to replicate. This can be done through changes in gene expression, or through direct physical contact with host machinery. With the advent of high-throughput techniques including transcriptomics, proteomics, yeast-two-hybrid (Y2H), affinity purification and MS (AP-MS), and lipidomics, there have been many advances in identifying these types of arbovirus-vector interactions.

### Gene Expression Profiling

A classic systems biology analysis of virus-host interactions includes understanding how gene expression at the RNA and protein level changes in response to a virus infection. Several groups have used transcriptomic and proteomic profiling to understand how gene expression patterns in these vectors change in response to arbovirus infection (Bonizzoni et al., 2012; Paradkar et al., 2015; Dong et al., 2017; Etebari et al., 2017; Saucereau et al., 2017; Xin et al., 2017; Shrinet et al., 2018). These unbiased approaches can identify factors critical to arbovirus replication. For example, Paradkar et al. used RNA-seq data as a starting point to demonstrate the role of Cul4 in promoting West Nile virus (WNV) replication in *Culex quinquefasciatus* cells (Paradkar et al., 2015). More recently, Xin et al. used quantitative global proteomic profiling in *Aedes albopictus* C6/36 cells to identify several cellular pathways to be perturbed by ZIKV infection, including innate immunity and the unfolded protein response (Xin et al., 2017). They further identified the ubiquitin-proteasome system as conserved hub for virus replication in both mosquito and mammalian cells. The authors used Bortezomib, a FDA-approved inhibitor of the 20S proteasome to inhibit ZIKV replication in both *Aedes aegypti* cells and mice. A similar proteomic profiling study of *Ixodes scapularis* ISE6 cells revealed hundreds of changes in protein abundance following Langat virus (LGTV) infection (Grabowski et al., 2016). Here, Grabowski et al. identified changes pathways related to metabolism, protein biosynthesis, and mTOR signaling, which could be targeted using chemical inhibitors. While the limited annotation of the *I. scapularis* genome restricts the insight that can be gleaned from pathway level analysis, this is a first of its kind proteomic study of flavivirus-tick interactions, and led to the subsequent identification of several LGTV replication factors in the tick host (Grabowski et al., 2017).

Mosquitoes are notorious for having highly repetitive genome sequences, and this can make sequence alignment for transcriptomic studies challenging even if assembled genomes have been available for some time. Moreover, rapid expansion of gene families resulting from highly active transposons mean that assigning gene function can also be challenging as duplicate genes rapidly evolve to develop novel functions. Some groups used transcriptomics coupled with proteomics to improve mass spectra identification in vectors with poorly annotated or unsequenced genomes. In a recent study of *A. aegypti* Aag2 cells, Maringer et al. used this technique to improve identification of active transposons and identify genomic regions with incomplete annotation (Maringer et al., 2017).

### Physical Interactions

Another type of arbovirus-vector interaction that can be probed using systems techniques is virus-host protein-protein interactions (PPIs). For example, two studies have identified PPIs between a DENV and *A. aegypti* proteins via Y2H screening (Mairiang et al., 2013; Tham et al., 2015). These studies have provided an initial landscape of flavivirus-vector PPIs, however flaviviruses proteins include many transmembrane domains. Alternative systems approaches, like the AP-MS approach used by Muñoz et al. to identify interactions between DENV membrane protein E and the *Aedes* vector (Muñoz et al., 2013), may be better suited to identifying such flavivirus-vector PPIs.

A key limitation for using AP-MS to identify arbovirus-vector PPIs is finding a system that allows for abundant viral protein expression and efficient vector protein identification. We found that codon optimization can help improve viral protein expression in cell lines derived from vectors with better proteome annotation. Using this strategy, we identified arbovirus-mosquito interactions for multiple DENV proteins directly in *A. aegypti* Aag2 cells using systematic AP-MS (Shah et al., 2018). In this study, we compared virus-host interactions between the human and mosquito hosts at the level of individual proteins, complexes and pathways. This multi-level analysis highlighted the Sec61 translocon as a conserved hub for flavivirus replication that could be pharmacologically modulated in both human and mosquito cells. In the future, systematic AP-MS will be a promising method for exploring the arbovirus-vector PPI landscape.

### Lipidomics

While gene expression profiling is limited by reference genome availability and quality, lipidomics analysis is not subject to the same limitation and represents an avenue for studying arbovirus-vector interactions in non-model vectors. In fact, Perera et al. executed one of the first “-omics” studies of arboviruses directly in a mosquito system by performing lipidomic analysis of DENV-infected *A. albopictus* C6/36 cells (Perera et al., 2012). Here, the authors demonstrate that lipids are broadly redistributed in mosquito cells in a way that may support specific aspects of DENV replication following infection, such as maintaining fluidity, bending these membranes to form replication complexes, and providing the negative curvature required for DENV-induced double-membraned vesicles that have been observed by electron microscopy (Welsch et al., 2009). Additional experiments performed by Chotiwan et al. in *A. aegypti* midgut tissue offer a comprehensive temporal view of lipid regulation *in vivo* for over 10 days following DENV infection (Chotiwan et al., 2018). In a first of its kind study, the authors confirmed many of the observations made in cell culture experiments, such as overall increases in glycerophospholipid content and perturbation of the sphingolipid biosynthesis pathway. However, the changes observed *in vivo* also proved to be more complex than those observed in cell culture. More recently, *Wolbachia*-infected *A. albopictus* Aa23 cells were shown to alter sphingolipid content in a direction opposite of what is observed during DENV infection, suggesting that *Wolbachia* infection may inhibit DENV infection through perturbation of lipid homeostasis (Molloy et al., 2016). Taken together, these studies highlight the utility of lipidomic analysis as an avenue for systems-level interrogation of arbovirus-vector interactions and underline the importance of translating global techniques to *in vivo* models.

## Understanding Vector and Arbovirus Evolution

Arboviruses replicate using error-prone polymerases and produce genetically diverse viral populations that facilitate their rapid evolution and adaptation to novel environments. Recent technological advances in sequencing can provide insight into how arbovirus-vector interactions impact arbovirus and vector evolution.

### Intra-Vector Viral Evolution

Arboviruses must overcome multiple bottlenecks in the vector to travel from the midgut to the salivary gland and be transmitted to a host. While these bottlenecks in mosquito were first observed using classical virology techniques (Smith et al., 2008), understanding the effects of these bottlenecks on virus evolution and fitness is possible because of the development of NGS technology that allows researchers to assess the diversity of viral populations over time and through different tissues on a large scale. For example, Grubaugh et al. used NGS and single variant analysis to track WNV diversity through the multiple bottlenecks in different mosquito vectors (Grubaugh et al., 2016b). The authors found that enzootic *Culex* vectors generated more intra-vector WNV diversity to overcome genetic drift associated with the transmission bottleneck compared to the *A. aegypti* bridge vector. Despite this diversity, the virus transmitted to avian systems by *Culex* vectors had lower relative fitness due to weak purifying selection that allows the accumulation of deleterious mutations. Similar studies exploring tick- and mosquito-borne virus intra-vector diversity suggest that diversity is determined by both the virus and the vector, and results from differences in the strength of purifying selection, bottleneck effects, and positive selection from antiviral responses (Stapleford et al., 2014; Brackney et al., 2015; Sim et al., 2015; Grubaugh et al., 2016a; Lequime et al., 2016; Patterson et al., 2018; Weger-Lucarelli et al., 2018). Further study will be needed to determine the impact of the different selective forces on viral genome evolution within their arthropod vector.

Long read sequencing, such as single-molecule real-time and nanopore sequencing, can provide additional information on viral diversity through the determination of recombination events and reconstruction of full-length genotypes. While long read technologies have a high error rate, multi-platform sequencing, which uses a combination of short and long reads, can compensate for this shortcoming. Jaworski and Routh used long and short read sequencing to detect recombination rates and determine wild type sequence frequency, which was essential to studying defective interfering RNA production in Flock House virus (Jaworski and Routh, 2017). Depledge et al. also used a multi-platform approach to analyze alternative splicing, transcription start sites and read through variants in viral transcripts (Depledge et al., 2018). While this approach was applied to herpes virus, it will be extremely valuable in the study of arbovirus-vector interactions, such as the impact of subgenomic flavivirus RNA on vector innate immunity and transmission (Göertz et al., 2016).

Another recent advance in NGS methodology, single cell RNA sequencing (scRNA-seq) (Tang et al., 2009), permits the study of how replication in specific cell types within various tissues contributes to viral diversity and transmission. A recent study by Severo et al. used the technique to characterize mosquito hemolymph cells (Severo et al., 2018). While this study was done in *Anopheles* mosquitoes, similar studies in arbovirus vectors will provide a foundational knowledge on vector immune cells. In a virus inclusive scRNA-seq analysis in human cells, Zanini et al. showed that DENV and ZIKV replication rates vary among cells and this variation correlates with differences in gene expression of host factors. The authors further used these correlations to identify host restriction and dependency factors (Zanini et al., 2018). Applying such scRNA-seq techniques to vector systems could provide insight into several open questions in the field of arbovirus-vector interactions, such as which vector pathways control viral replication and transmission in different tissues, and which cell types control viral persistence, vertical transmission, and the selection of viral variants *in vivo*. In this way, scRNA-seq provides a unique opportunity to bridge the study of intracellular arbovirus-vector interactions with viral evolution, immunity and transmission *in vivo*.

### Long-Term Evolutionary Interactions

In addition to direct sequencing of viral populations, arthropod genome sequencing projects enabled by NGS technologies have improved the ability to understand long-term interactions between arboviruses and vector genomes. Endogenous viral elements (EVEs) are viral sequences that are inserted into the host genome. EVEs inserted into the germline can provide a record of past infections and may provide a source of antiviral immunity. Consequently, in contrast to arbovirus intra-vector evolution, arbovirus-derived EVE integration events reflect the long-term evolutionary relationship between arboviruses and their arthropod vectors.

The organization, evolution, and mode of action of EVEs in arthropod genomes are still poorly understood, but their characterization has benefited from progress in arthropod genomics. EVE integration occurs mainly in Piwi-interacting RNA (piRNA) clusters, genomic regions known to be composed of incomplete transposon sequences and a source of piRNAs production. piRNA are the main defense system against transposition in many species (Aravin et al., 2007) and described as a potential antiviral defense for mosquito (Miesen et al., 2016). Using small RNA NGS to profile piRNA production from EVEs, several studies have found the production of anti-sense piRNAs mostly restricted to EVEs in piRNA clusters (Palatini et al., 2017; Suzuki et al., 2017; Whitfield et al., 2017). This piRNA production suggests a role for EVEs in the antiviral response against new infection of arthropods, a hypothesis that is supported by a recent study that observed a difference in piRNA production during DENV infection of *Aedes* mosquitoes (Wang et al., 2018).

In addition to providing insights into EVE biogenesis, NGS techniques can also help identify more EVEs through the generation of new arthropod genomes to survey and improvements in genome assemblies. For example, while arthropod EVEs described in the literature belong to many different virus families, a comprehensive metagenomic study of 48 arthropod genomes identified over 4000 EVEs, and found that most belong to *Rhabdoviridae* and *Parvoviridae* families (ter Horst et al., 2018). Interestingly, no EVEs from *Togavirirdae* were found in this or other studies, even though family members like CHIKV are major arboviruses. Finally, Whitfield et al. used long read sequencing technology to increase the resolution of highly repetitive regions of *A. aegypti* Aag2 cell genome, allowing the discovery of unknown EVEs from the *Rhabdoviridae, Flaviviridae*, and *Chuviridae families* (Whitfield et al., 2017). This study suggests that the arthropod EVE population is underestimated, and also highlights the utility of new sequencing technologies for increasing our genomics resolution to aid in EVE discovery.

## Mapping Arthropod-virome Interactions

The emergence of ZIKV has reignited concern about identifying arboviruses that pose a risk for emergence. Due to the declining cost and increasing portability of NGS technologies, researchers can now identify new arboviruses, determine their range, and understand vector competence, all of which can contribute to the arboviral emergence.

### Arbovirus Discovery

There is great interest in identifying arboviruses in field-caught vectors, and several groups have now sequenced hundreds or even thousands of field-caught mosquitoes and ticks to survey the vector virome (Tokarz et al., 2014; Frey et al., 2016; Harvey et al., 2018; Sadeghi et al., 2018; Zakrzewski et al., 2018). Together, these studies have identified dozens of new arboviruses. In the future, virome sequencing could be especially useful for surveillance, tracking the spread of pathogenic viruses. The spread of low-cost nanopore sequencing represents an interesting virus discovery tool, as the portable MinION was used to identify arboviruses in field-caught mosquito (Russell et al., 2018). In the future, these tools will advance virus discovery in understudied vectors like the sandfly.

### Vector Competence

scRNA-seq can also be used inform on complex topics like vector competence. Several recent studies have shown that the presence of arthropod-specific viruses can inhibit or promote the replication of medically-relevant arboviruses (Goenaga et al., 2015; Nasar et al., 2015; Schultz et al., 2018) and could impact vector competence. Co-infection with bacteria like *Wolbachia* species can also reduce vector competence (Glaser and Meola, 2010). Virome and microbiome mapping of an individual vector is the first step in relating infection status to vector competence. Recently, Cross et al. commenced such an effort by coupling virome and microbiome analysis in individual *I. scapularis* ticks (Cross et al., 2018). The authors found a positive and negative correlations correlation between different arboviruses, and between levels of specific tick-borne virus RNA and co-infections with *Borrelia burgdorferi*, the bacterium that causes Lyme disease. These results support the current model that the virome and microbiome influence vector competence and highlight the need for more studies in this area.

## Caveats and Potential Solutions

Despite the advances made using systems biology approaches to study arbovirus-vector interaction, several challenges remain. First, the lack of reference genomes for many vectors limits the systems techniques that can be applied to them. However, the i5K initiative to sequence 5,000 arthropod genomes (Poelchau et al., 2018) will fill critical gaps in this area. Poor genome annotation also limits the utility of systems biology studies, and further development of methods to predict gene function will be important for progress. Finally, as with all systems biology studies, improvements in data collection enabled by systems biology approaches need to be accompanied by advances in techniques capable of transforming that information into mechanistic insight. Better genetic tools like the recent advances in heritable CRISPR genome editing of arthropods (Chaverra-Rodriguez et al., 2018) will enable rapid testing of specific arthropod genes that interact with arboviruses for their impact on virus replication *in vivo*. Ultimately, these tools could be used for arbovirus mitigation efforts. For example, CRISPR could be used to target non-essential arbovirus replication genes to engineer arthopod populations with reduced vector competence without compromising overall arthropod fitness.

## Conclusions

Systems biology approaches offer global, unbiased views of arbovirus-vector interaction landscapes. By taking advantage of these approaches, researchers have the potential to transform our understanding of arbovirus replication, evolution, diversity, and vector competence. In the long-term, this will offer insight into the basic biology of arboviruses and their vectors, and lead to the development of arbovirus mitigation strategies.

## Author Contributions

MP and PS conceived of topics to be discussed and wrote the manuscript. MP wrote sections pertaining to virus and vector evolution. PS contributed to all sections of the manuscript.

### Conflict of Interest Statement

The authors declare that the research was conducted in the absence of any commercial or financial relationships that could be construed as a potential conflict of interest.
